# Supplementary figures and images for: Single molecule analysis of CENP-A chromatin by high-speed atomic force microscopy
Source: eLife. 2023 Sep 20;12:e86709. doi: 10.7554/eLife.86709 (PMC10511241; doi:10.7554/eLife.86709)

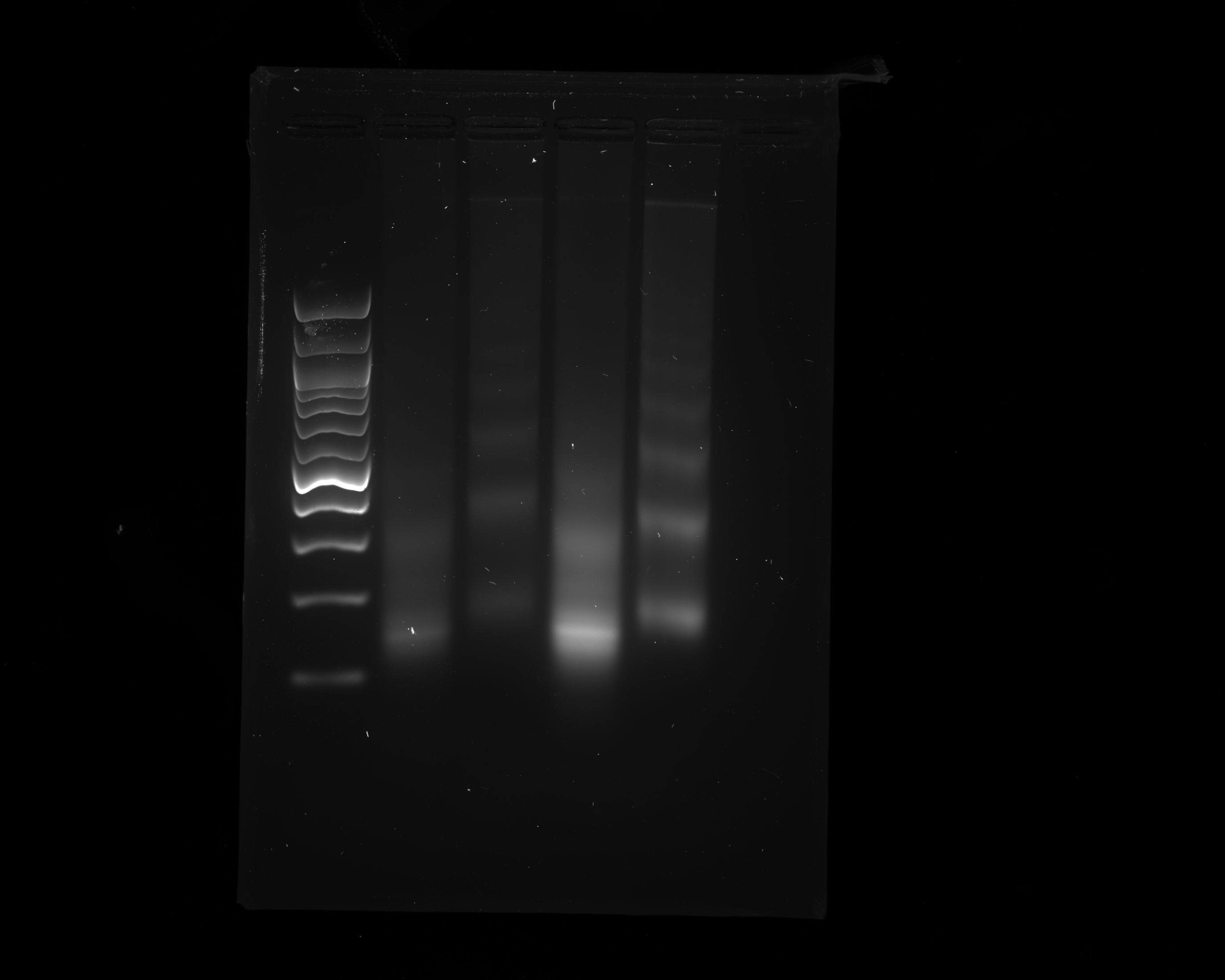

Supplement: Figure 1—figure supplement 1—source data 1. [file elife-86709-fig1-figsupp1-data1.zip › Figure_Supplement_1-Source_Data_1/CSEM_MP 2019-06-07 16h35m16s(GelStar).raw16.tif]

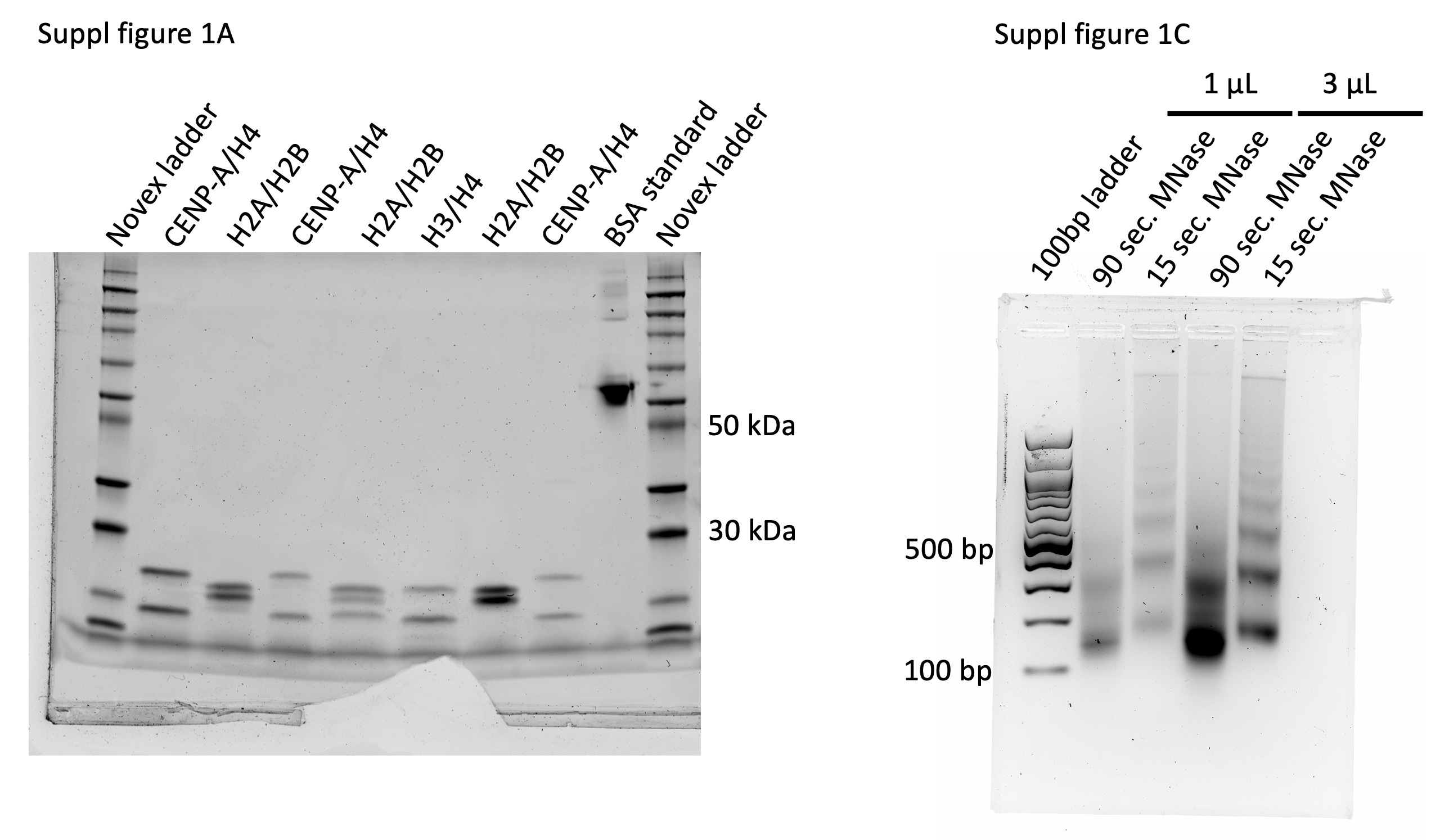

Supplement: Figure 1—figure supplement 1—source data 1. [file elife-86709-fig1-figsupp1-data1.zip › Figure_Supplement_1-Source_Data_1/Supplemental_figure_1_raw_image_annotated.tiff]

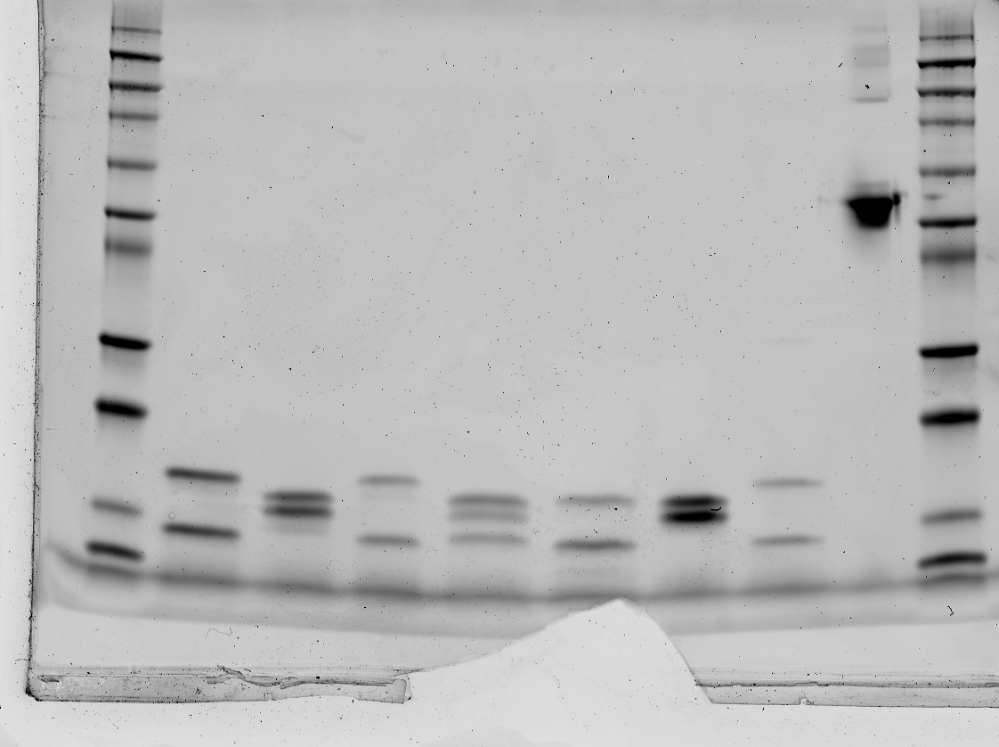

Supplement: Figure 1—figure supplement 1—source data 1. [file elife-86709-fig1-figsupp1-data1.zip › Figure_Supplement_1-Source_Data_1/Coomassie_gel_histones_raw_image.tiff]

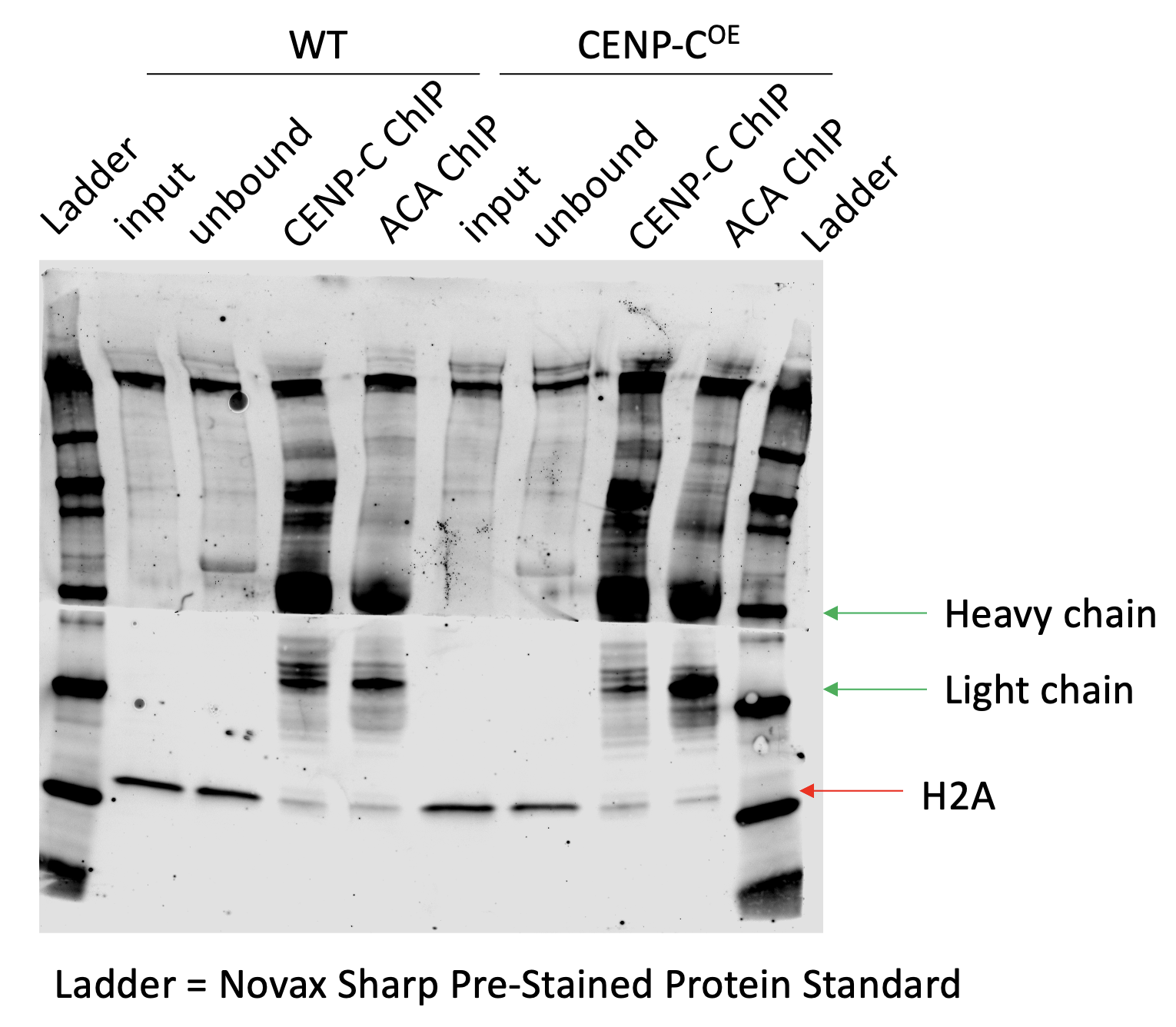

Supplement: Figure 5—figure supplement 1—source data 1. [file elife-86709-fig5-figsupp1-data1.zip › Figure_5-Figure_Supplement_16-Source_Data_6/Supplement_Figure_16b-Source_Data_6.tiff]

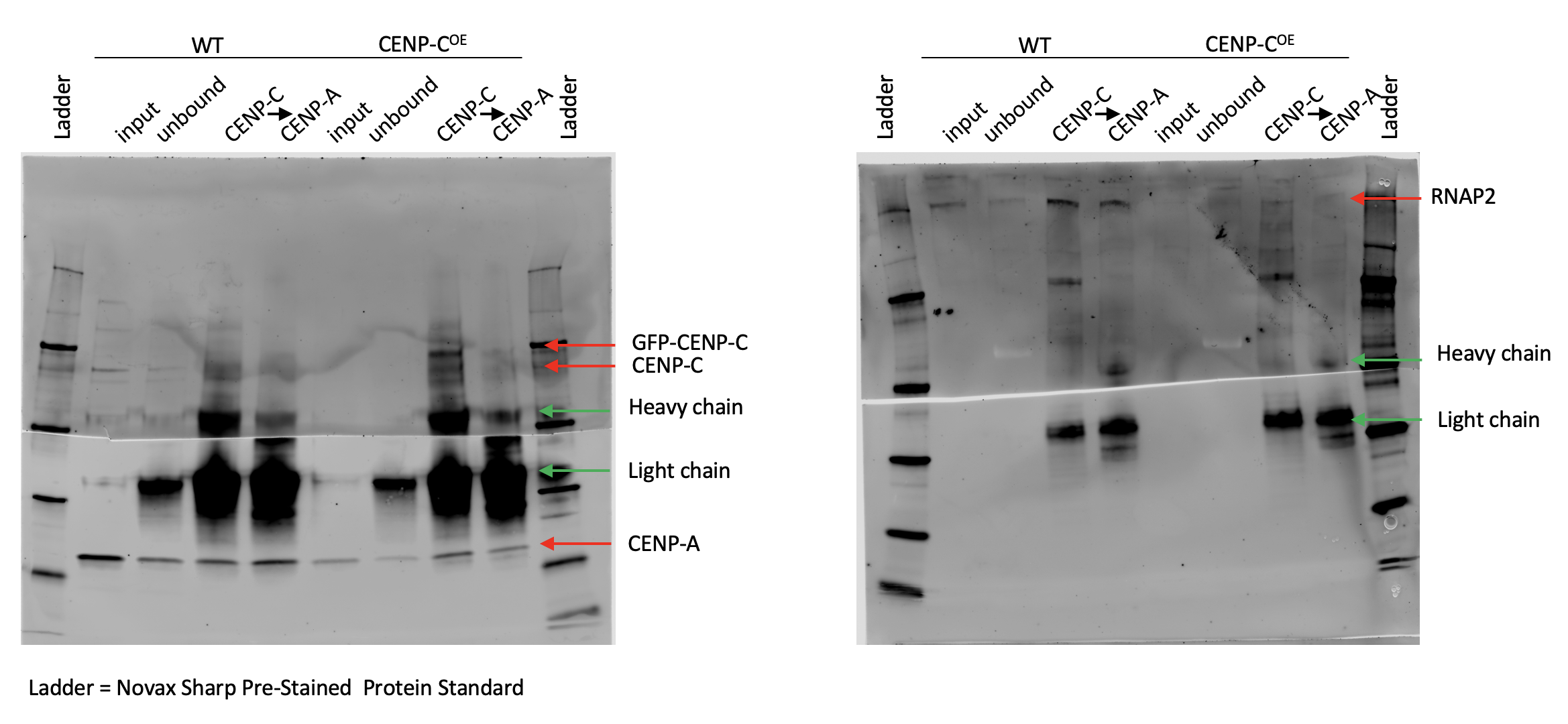

Supplement: Figure 5—figure supplement 1—source data 1. [file elife-86709-fig5-figsupp1-data1.zip › Figure_5-Figure_Supplement_16-Source_Data_6/Supplement_Figure_16a-Source_Data_6.tiff]

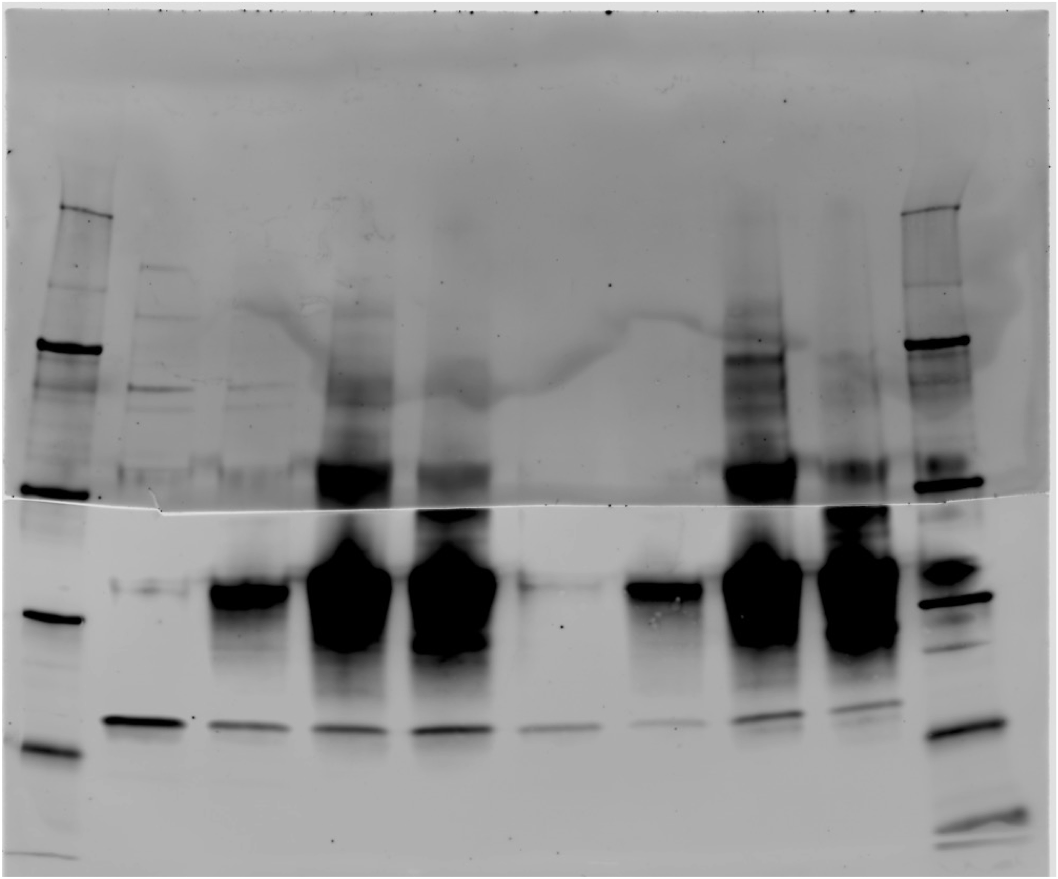

Supplement: Figure 5—figure supplement 1—source data 1. [file elife-86709-fig5-figsupp1-data1.zip › Figure_5-Figure_Supplement_16-Source_Data_6/Western_blot_raw_image1.tiff]

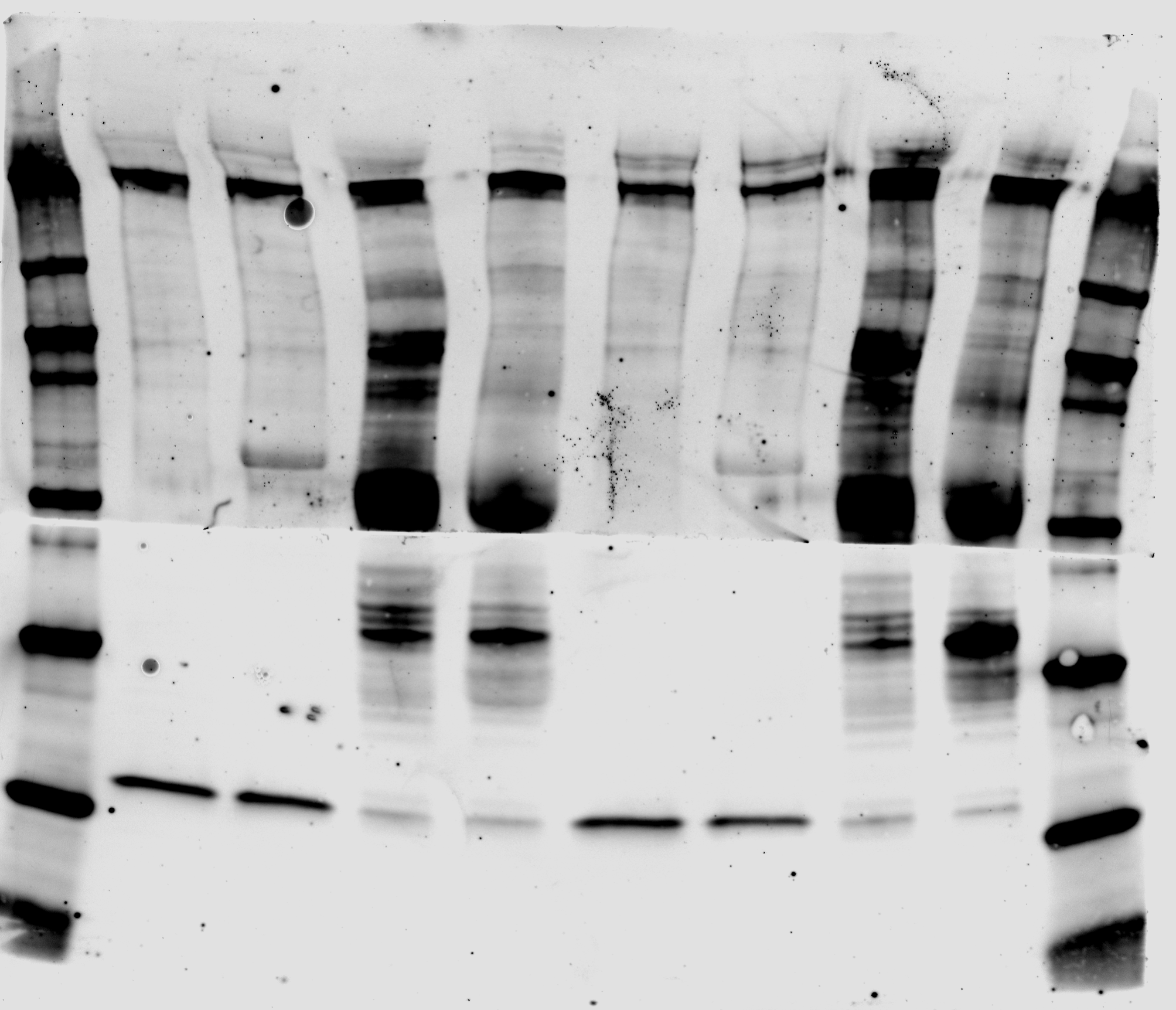

Supplement: Figure 5—figure supplement 1—source data 1. [file elife-86709-fig5-figsupp1-data1.zip › Figure_5-Figure_Supplement_16-Source_Data_6/Western_blot_raw_image3.tif]

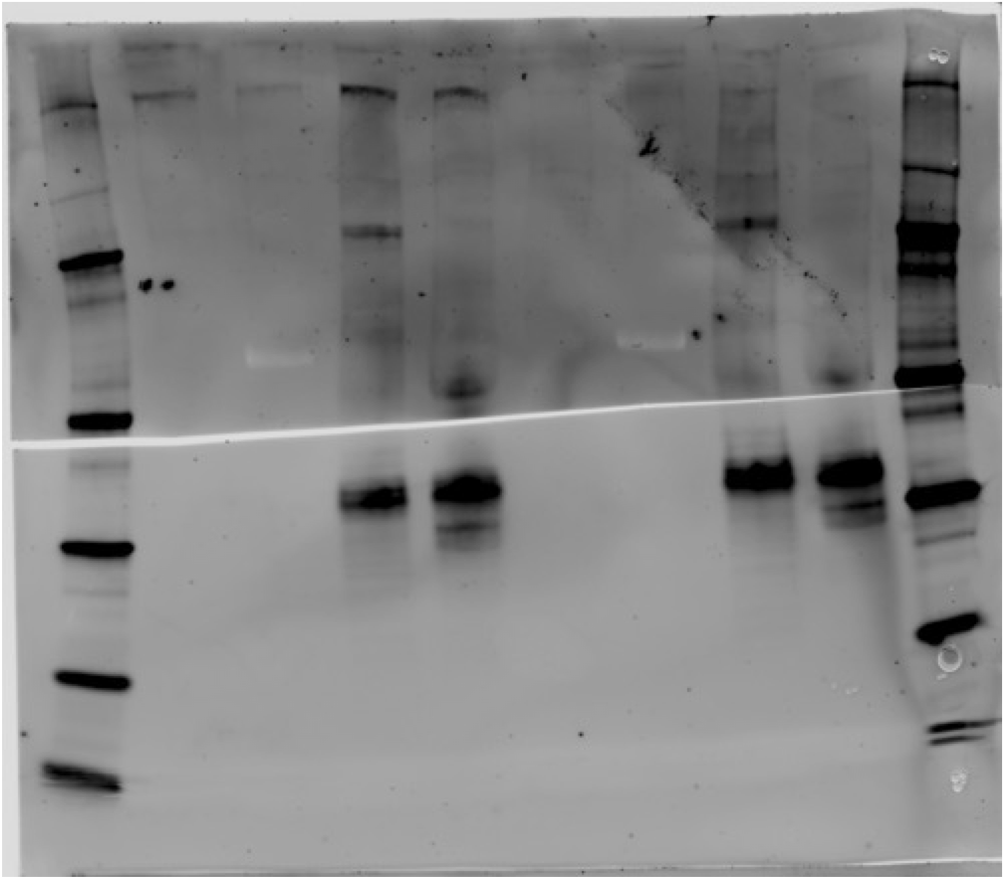

Supplement: Figure 5—figure supplement 1—source data 1. [file elife-86709-fig5-figsupp1-data1.zip › Figure_5-Figure_Supplement_16-Source_Data_6/Western_blot_raw_image2.tiff]
